# Phenotype of regulatory T cells in human type 1 diabetes at diagnosis and partial remission phase

**DOI:** 10.1186/1758-5996-7-S1-A208

**Published:** 2015-11-11

**Authors:** Daniela da Silva Camilo, Adriel Santos Moraes, Fernando Pradella, Paula Giovana Russini, Alliny Carolina Dionete Lima, Ana Leda Longhini, Maria Fernanda Vanti Macedo Paulino, Sofia Helena Valente de Lemos Marini, Gil Guerra, Elizabeth João Pavin, Candida Parisi, Alessandro dos Santos Farias, Leonilda Maria Barbosa dos Santos, Walkyria Mara Gonçalves Volpini

**Affiliations:** 1 UNICAMP, Brazil

## Background

Human type 1A diabetes (T1AD) has a broad spectrum of clinical presentations, which may be associated with the severity of autoimmune response and consequently, different levels of pancreatic beta cells destruction. The T1AD presents a partial remission phase. The remission phase is classically a short period in childhood-onset diabetes, but longer periods may occur especially in young.

## Objective

This study was designed to investigate cellular immunity focusing regulatory T-cells (Tregs) in different disease stages of the disease.

## Materials and methods

A total of 13 T1AD patients: 8 newly-diagnosed T1AD (age: 7.9±6.3 yrs., insulin dose: 0.5 U/kg/day) within 1.0±0.9 months of their diagnosis, 5 in partial remission, for 1.2±1.0 yrs. after diagnosis (age: 10.8±6.8 yrs., insulin dose: 0.2 U/kg/day) and 9 healthy controls (21.9±2.7yrs.) were studied. Phenotypic analysis of Tregs was performed by flow cytometry on peripheral blood. After a Lyse/Wash protocol, cells were stained for CD4, FoxP3, CTLA4, CD25. T cell markers CD25, CTLA-4 and FoxP3 were examined on cells within the CD4 gate. Groups were compared using an one-way ANOVA test.

## Results

The frequency of circulating CD4+CD25+ and CD4+FoxP3+ T cells was significantly reduced in newly-diagnosed T1AD compared to patients in partial remission and controls (1.7±0.6% vs 4.0±2.1% vs 3.3±1.2%, p<0.01 and 0.7±0.7% vs 2.0±2.0% vs 2.3±0.8%, p<0.03 respectively).

## Conclusions

These preliminary data showed decreased peripheral Tregs frequency in classical childhood-onset T1AD. In contrast, the group of long-term remission patients had similar frequency to controls and some of them presented latent autoimmune diabetes features. Immunophenotyping at the time of diagnosis and during follow-up may help the definition of both T1AD clinical subtypes and remission period.

